# Survey of the Intermolecular Disulfide Bonds Observed in Protein Crystal Structures Deposited in the Protein Data Bank

**DOI:** 10.3390/life12070986

**Published:** 2022-06-30

**Authors:** Oliviero Carugo

**Affiliations:** 1 Department of Chemistry, University of Pavia, 27100 Pavia, Italy; oliviero.carugo@univie.ac.at; 2 Italy & Max Perutz Labs, Department of Structural and Computational Biology, University of Vienna, 1010 Wien, Austria

**Keywords:** intermolecular interaction, disulfide bond, post-translational modification, Protein Data Bank, protein structure

## Abstract

About 5% of the disulfide bonds (DBs) observed in the Protein Data Bank bridge two protein chains. Several of their features were comprehensively analyzed, resulting in a structural atlas of the intermolecular DBs. The analysis was performed on a very large set of data extracted from the Protein Data Bank, according to the RaSPDB procedure. It was observed that the two chains tend to have different sequences and belong to the same structural class. Intermolecular DBs tend to be more solvent accessible and less distorted from the most stable conformation than intermolecular DBs while showing similar B-factors. They tend to occur in beta strands and in mainly-beta structures. These and other data should prove useful in protein modelling and design.

## 1. Introduction

Disulfide bonds (DBs) are amongst the most studied post-translational modifications of proteins. Discovered more than a century ago by Heffter (in 1907) and Arnold (in 1911), during studies on the presence of -SH groups in coagulated egg albumin [cited by [1]], the mechanism of their formation was clarified later, with the discovery of the protein disulfide isomerase in eukaryotes [2,3] and of the thiol disulfide oxidoreductase DsbA in prokaryotes [4], two enzymes that catalyze the -SH groups’ oxidation to disulfide.

The structural features and roles of intermolecular DBs were reviewed by Thornton [5], who observed that “there is a strong preference for shorter connections, with half-cystines separated by fewer than 24 residues in 49% of all disulfides”. Several subsequent papers have analyzed disulfide bonds in many ways: the role of disulfides in protein oxidative folding and assembly [6,7,8,9], disulfides’ design [10], disulfide-related protein sorting [11,12], reaction mechanisms of disulfide formation in cells [13], role of DBs on protein stability [14] and stability of vicinal disulfides [15], amongst others.

However, most of these studies focused on *intramolecular* DBs, while structural and functional features of *intermolecular* DBs have received little attention. This is not surprising since intramolecular DBs are more frequent than intermolecular DBs. However, intermolecular DBs have a central importance in several biological processes.

Intermolecular DBs are important for innate immunity. For example, the human antimicrobial peptide LL-37_17−29_ forms intermolecular bridges and then a thermostable fibrillar structure of densely packed helices that surround bacterial cells [16]. It has been speculated that this is a general mechanism for short, helical antimicrobial peptides since they contain an odd number of cysteines, one of which is prone to form reversible intermolecular DBs [16]. The formation of intermolecular DBs between LL-37 and bovine serum albumin is believed to stabilize protein nanoparticles that might be used as promising vehicles to deliver therapeutics, otherwise unstable, for the treatments of lung infections from *Pseudomonas aeruginosa* [17].

Two cysteine residues of the *Staphylococcus aureus* catabolite control protein A (CcpA) have been shown to be important for resisting human innate immunity, too: they can be oxidized and form an intermolecular DB between two CcpA dimers, with the formation of a tetramer and CcpA dissociation from its cognate DNA promoter [18].

Protein aggregation and amyloidogenesis, which are related to a multitude of so-called conformational diseases, are influenced by DB formation: it has been shown that globular, native structures tend to be stabilized by intramolecular DBs, while intermolecular DBs may favor aggregation [19]. For example, bovine milk α_S2_-casein forms amyloid fibrils in vitro and is probably involved in the formation of fibrils in vivo in mammary tissue. It is either monomeric, when its two cysteines form an intramolecular DB, or dimeric, with two intermolecular DBs. The dimeric form has a greater propensity to form fibrils than the monomeric form [20]. The aggregation of β-parvalbumin is initiated by the formation of dimers stabilized by intermolecular DBs [21].

Fonin and colleagues showed that intermolecular DBs between promyelocytic leukemia protein (PLM) monomers are required for the formation of PLM nuclear bodies [22]. Moreover, basic helix–loop–helix leucine zipper transcription factors play a relevant role in mediating the cellular response to several stress conditions, such as oxidative stress and nutrient deprivation. Interestingly, oligomers of these proteins, stabilized by intermolecular DBs, are considerably more stable under protracted stress conditions [23].

Flores-Solis and colleagues proposed that the solvent-exposed cysteines of EhICP1, an inhibitor of cysteine protease 1 from *Entamoeba histolytica*, in oxidative conditions, form intra- and intermolecular DBs that render an inhibitor inactive [24]. Moreover, regulation of neurexins, presynaptic adhesion molecules, occurs with the formation of intermolecular DBs with protein FAM19A1-A4 [25]. The oligomerization degree of a secreted hormone with metabolic function, myonectin—from trimers to hexamers and to high-molecular-weight oligomers—depends on the formation of intermolecular DBs [26]. Designed intermolecular DBs have been used to tune the internal dynamics of dimeric proteins [27]. Oligomerization is induced by intermolecular DBs in proteins of the photoreceptor outer segments: peripherin 2 and its homologue Rom1 form non-covalent homo and hetero tetramers that assemble into higher-order complexes, held together by intermolecular DBs [28].

In the present article, I review the structural features of the intermolecular DBs observed in the protein three-dimensional structures deposited in the Protein Data Bank [29,30]. The analysis is divided into three parts.

The first is focused on general features of the intermolecular DBs: the frequency of intermolecular DBs is compared to the frequency of intramolecular DBs and the relative frequencies of homo- and heteromeric intermolecular DBs are determined.

In the second part, local features of the intermolecular DBs are described and they are compared to those of the intramolecular DBs, namely their secondary structures and solvent accessibility, the stereochemistry of the S-S bond and the B-factors of the sulfur atoms.

Finally, global features of proteins that contain intermolecular DBs are described and compared to those of proteins that contain intramolecular DBs, namely the fold types and, in the case of enzymes, the types of enzymes.

This survey of the Protein Data Bank’s intermolecular DBs can be viewed as a kind of anatomical atlas, which will need periodic updates as new information becomes available, and which should prove useful in protein modeling and engineering.

## 2. Methods

### 2.1. Data Selection

All data were taken from the Protein Data Bank [29,30]. The analysis was limited to protein X-ray crystal structures. Multi-model refinements and structures containing only Cα atoms were discharged. Only structures refined at a resolution better than 2.5 Å were retained, and this resulted in a list of about 121,000 entries of the Protein Data Bank.

These were randomly divided into 14 subsets, each containing 7000 entries. Each entry was contained in one subset only (no overlap). All the analyses were then performed on each of the 14 subsets.

This procedure, named *RaSPDB*, was proposed recently to enlarge data coverage in statistical surveys of the Protein Data Bank [31].

Traditionally, *redundancy* is the prominent criterion for assembling statistically significant subsets of the Protein Data Bank [32]. This is justified by the fact that it is well known that the Protein Data Bank is quite redundant and redundancy is usually reduced at the sequence level, by rejecting proteins that are too homologous.

However, it has been shown that one can build several subsets of the Protein Data Bank and that all of them can be used to estimate statistically significant structural trends —for example the frequency of DBs—disregarding sequence redundancy. This is due to the fact that the Protein Data Bank is extremely large nowadays so that these subsets can be (i) sufficiently large to represent the entire Protein Data Bank, and (ii) sufficiently small to prevent a too extensive inter-subset redundancy.

This procedure has two main advantages. On the one hand, it allows the use of more information stored in the Protein Data Bank; on the other hand, it allows the estimation of the standard deviations of the structural trends extracted from the Protein Data Bank: in fact, if a trend is estimated *n* times with *n* subsets, one can analyze the distribution of these *n* estimations.

### 2.2. DB Identification

DBs can be identified in protein three-dimensional structures by computing the sulfur–sulfur bond length that must be close to 2 Å. Alternatively, the Protein Data Bank files report explicitly the DBs in specific lines that begins with the “SSBOND” label. These two procedures provide slightly different results since some of the DBs listed in the SSBOND files are reduced, at least partially, during X-ray diffraction experiments and, consequently, the DB is broken, at least partially, in the crystal.

For this reason, both procedures were used here.

On the one hand (*FIND* method), DBs were detected from the atomic coordinates, under the condition that the S-S covalent bond length is in the 1.9–2.1 Å range (in case of conformational disorder, only the first conformation was considered).

On the other hand (*GREP* method), all the DBs listed in the “SSBOND” lines of the PDB-formatted files were taken into consideration.

More details on the FIND and GREP procedures are available in the Supplementary Materials.

### 2.3. Miscellaneous

Secondary structures were assigned with Stride [33] and DSSP [34,35]. Solvent accessible surface areas were computed with NACCESS [36]. B-factors were normalized to zero mean and unit variance by means of

(1) $$BN=\frac{B-Bave}{Bstd}$$

where *Bave* is the average *B*-factor of the protein and *Bstd* is its standard deviation [37].

## 3. Results and Discussion

### 3.1. General Features

#### 3.1.1. How Frequent Are Intermolecular DBs?

Although it is known—*qualitatively*—that intermolecular DBs are rather infrequent, a *quantitative* estimation of their frequency of occurrence is provided here: about 4.5% (±0.3%) of the DBs are intermolecular and this value ranges from 3.7% to 5.6%, amongst the 14 subsets of the Protein Data Bank, if DBs are detected by the FIND method. Similar values (average = 4.8% (±0.3%) and range from 4.1% to 4.6%) are observed if DBs are detected by the GREP method. Additional details are provided in the Supplementary Materials (Table S1).

This indicates that the majority of the DBs are intramolecular. It is not surprising that intermolecular DBs are rather uncommon since the correct protein folding—both in vitro and in vivo—can be problematic when a covalent interaction must be formed between two independent macromolecules. However, intermolecular DBs are not rarities.

#### 3.1.2. Homomeric and Heteromeric Intermolecular DBs

The two protein chains connected by an intermolecular DB tend to be different (68% (±1%) if the DBs are identified with the FIND method and 61% (±1%) if the DBs are identified with the GREP method), and these values range from 59% to 76% (FIND method) and from 56% to 68% (GREP method) in the 14 subsets of the Protein Data Bank that are examined here. Additional details are provided in the Supplementary Materials (Table S2).

This is rather surprising because of the exponential growth of the number of different intermolecular DBs that can be formed when the number of different proteins increases. For example, if there is only one protein (P-SH), only one type of intermolecular DB can be formed (P-SS-P); if there are two proteins (P-SH and R-SH), three types of intermolecular DBs can be formed (P-SS-P, R-SS-R and P-SS-R); and if there are N proteins, N(N + 1)/2 types of intermolecular DBs can be formed.

The fact that intermolecular DBs connect different proteins clearly indicates the existence of a highly efficient regulation and control of protein folding and assembly in vivo.

#### 3.1.3. Number of Intermolecular Connections

The number of disulfide interconnections between protein chains was examined, too (Table 1). In most of the cases, only one intermolecular DB is present between two protein chains. Only about 15–20% of the protein chains are connected through two or more DBs. This seems to be slightly more frequent if the two protein chains are different. Additional details are provided in the Supplementary Materials (Table S3).

The presence of few, generally only one, intermolecular DBs is expected because of the principle of maximal simplicity that pervades biological molecules—the famous Newtons’ principle: nature is pleased with simplicity.

The observation that the number of intermolecular DBs tends to be slightly larger when the two proteins are different supports the hypothesis mentioned above: a mechanism to prevent misassembled DBs must exist and work on a variety of systems.

#### 3.1.4. Amino Acid Composition

The percentages of cysteines were computed for several groups of proteins that contain DBs. They are higher for proteins that contain both intra- and intermolecular DBs and lower for proteins that contain only intermolecular DBs.

On average, the percentage of cysteines is 4.32% (±0.08) for proteins that have both intra- and intermolecular DBs detected with the FIND method (4.28% ± 0.09 if DBs are detected with the GREP method).

These values are smaller (3.00% ± 0.02 and 2.91% ± 0.02) for proteins that contain only intramolecular DBs and even smaller (2.14% ± 0.10 and 1.86% ± 0.06) for proteins that contain only intermolecular DBs.

These differences are likely to be significant since the distributions of the percentages are not superposed. In fact, the percentage of cysteines ranges from 3.69% to 4.73% (FIND method) and from 3.55% to 4.28% (GREP method) amongst the 14 subsets of the Protein Data Bank for proteins that contain both intra- and intermolecular DBs; from 2.83% to 3.14% (FIND method) and from 2.76% to 3.03% (GREP method) for proteins that contain only intramolecular DBs; and from 1.69% to 2.94% (FIND method) and from 1.56% to 2.34% (GREP method) for proteins that contain only intermolecular DBs.

### 3.2. Local Features

#### 3.2.1. Secondary Structures

The secondary structures of the cysteines that form intra- and intermolecular DBs were compared. Table 2 shows the most relevant observations.

DBs tend to be formed between cysteines in strands (EE in Table 2) This tendency is more marked, however, for intramolecular than for intermolecular DBs. Intermolecular DBs are frequently formed when the two cysteines are in helices, while they are very rare when one cysteine is in a helix and the other in a strand. Additional details can be found in the Supplementary Materials (Table S4).

There are minor differences between the secondary structure assignments made with DSSP and Stride: DBs between cysteines in turns are quite common with Stride assignments and rare with DSSP assignments. These discrepancies are rather surprising since it is common to use either DSSP or Stride for secondary structure assignments, assuming that these two programs are equivalent. In reality, they have different algorithmic bases and, consequently, can produce different assignments.

There are, therefore, both similarities and differences between the secondary structures of intra- and intermolecular DBs, suggesting that they might have different evolutionary histories and different roles.

#### 3.2.2. Solvent Accessibility

Intramolecular DBs tend to be well buried onto the protein core. The average solvent accessible surface area value (SASA) of the cysteines that form them is only 11.4 (±0.1) Å^2^ if disulfides are identified with the FIND method or 11.6 (±0.1) Å^2^ if they are identified with the GREP method.

Intermolecular DBs tend to be slightly more solvent exposed. The average SASA of the cysteines that form them is 24.4 (±0.1) Å^2^ if DBs are identified with the FIND method or 27.2 (±0.1) Å^2^ if they are identified with the GREP method.

Additional details for the 14 subsets of the Protein Data Bank examined in the present paper are available in the Supplementary Materials (Table S5).

**Figure 1:**
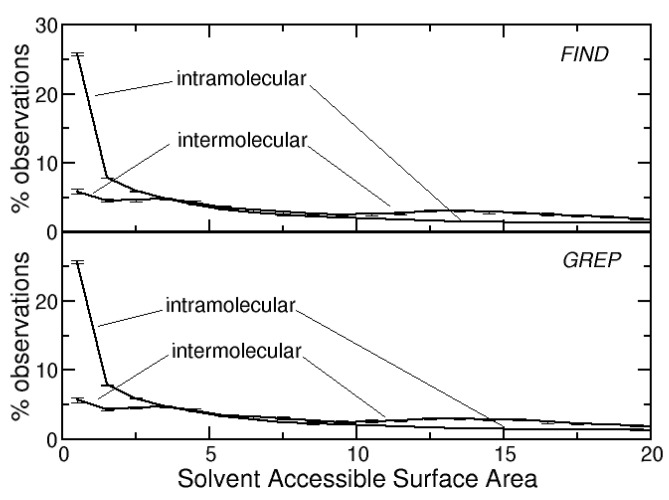
Distribution of the solvent accessible surface areas (Å^2^) for intra- and intermolecular DBs identified with the FIND or the GREP methods.

The distributions of the SASAs of the cysteines that form DBs are shown in Figure 1. They are much more skewed towards zero for the intramolecular DBs and more spread for intermolecular DBs. About 25% of the cysteines that form intramolecular DBs have a SASA lower than 1 Å^2^. This percentage is about five times smaller for cysteines that form intermolecular DBs

#### 3.2.3. Stereochemistry

**Figure 2:**
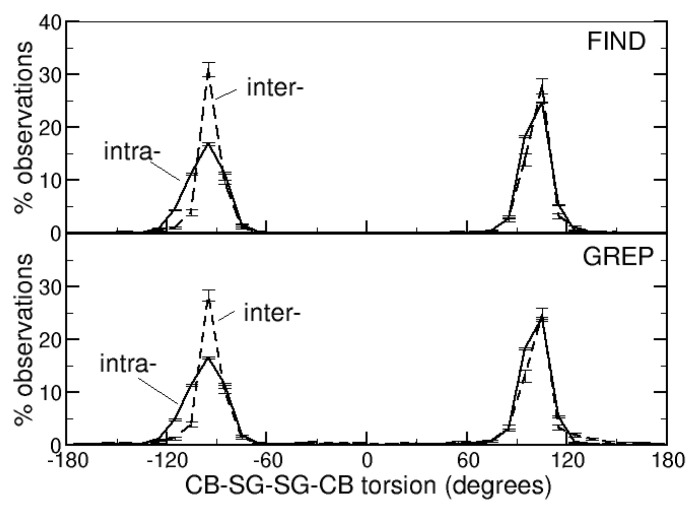
Distribution of the torsions defined by the CB-SG-SG-CB atoms of the side-chains of the cysteines that form the intra- or intermolecular DBs, identified with the FIND or GREP method.

The torsions defined by the CB-SG-SG-CB atoms of the side-chains of the cysteines that form intra- or intermolecular DBs have been measured. Their distributions (Figure 2) are similar: there are two maxima, one around −95° and the other around 105°. There are, however, subtle differences; the peaks are sharper for intermolecular DBs. In the range (−90°, −100°), there are 31% or 28% of observations for intermolecular DBs (identified with the FIND and GREP methods, respectively) and only 17% or 16% for intramolecular DBs (FIND and GREP, respectively). This trend is observed also in the range (100°, 110°), although less markedly: 28% and 25% of observations for intermolecular DBs (FIND and GREP, respectively) and 25% and 24% for intramolecular DBs (FIND and GREP, respectively). Additional details for each of the 14 subsets of the Protein Data Bank are given in Figure S1 (Supplementary Materials).

The CB-SG-SG-CB torsions of intra- and intermolecular DBs show, therefore, the same tendencies, but intermolecular DBs seem to be less distorted from the rotameric conformation. This might be related to their solvent accessibility, which is larger, on average, than that of the intramolecular DBs. Being more solvent exposed, they make a lower number of interactions with other protein atoms that can cause distortions from their rotameric conformations.

#### 3.2.4. B-Factors

The normalized B-factors were computed and each DB was associated with the average normalized B-factor of its two sulfur atoms. Intra- and intermolecular DBs have very similar normalized B-factors (0.034 (±0.006) and 0.089 (±0.028), respectively, for DBs identified with the FIND method, and 0.044 (±0.009) and 0.093 (±0.021), respectively, for DBs identified with the GREP method).

**Figure 3:**
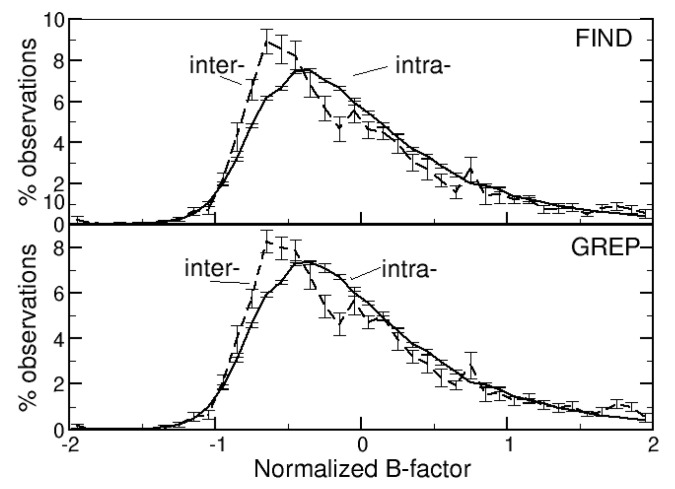
Distribution of the normalized B-factors of the sulfur atoms of the side-chains of the cysteines that form the intra- or intermolecular DBs, identified with the FIND or GREP method.

The distributions of the normalized B-factors are very similar for intra- and intermolecular DBs, too (Figure 3 and Figure S1, Supplementary Materials). They are positively skewed and, consequently, their maxima are well below BN = 0, though a few values <−1 are observed.

### 3.3. Global Features

#### 3.3.1. Enzyme Types

Interesting trends were observed by investigating the distribution of intra- and intermolecular DBs in the seven types of enzymes (Table 3; disaggregated data for each of the 14 subsets of protein structures examined in the present paper are given in the Supplementary Materials, Table S6).

About 40–50% of the proteins that contain DBs—only intramolecular, only intermolecular or both—are enzymes, and nearly all of them are hydrolases when intramolecular DBs are present; when there are only intermolecular—and no intramolecular—DBs, different types of enzymes can be observed—mainly oxidoreductases, transferases and hydrolases but also, with lower frequency, lyases, isomerases and ligases.

It is thus reasonable to hypothesize that the formation of intermolecular DBs, in enzymes that do not contain intramolecular DBs, is a general mechanism to stabilize their structure and, consequently, to confer a wide variety of functionalities. In other words, it is a mechanism that works for any type of enzymes. Notably, the frequency of enzymes containing only intermolecular DBs is similar to the frequency of the same type of enzymes in the Protein Data Bank (https://www.rcsb.org/stats/explore/enzyme_classification_name, accessed on 30 June 2022; Table S7 in the Supplementary Materials).

#### 3.3.2. Structural Classes

The structural classes of the proteins involved in DBs were identified by using the Scop database [38,39], which is not only a collection but also a hierarchical classification of protein structural domains. At the top level there are four classes: *all-alpha* (a), *all-beta* (b), *alpha-and-beta* (c), and *alpha-plus-beta* (d). The second level of classification is the fold, and then other levels follow with increasing degrees of homology and sequence similarity.

Other classes, even if considered in Scop, are not taken into consideration here since they comprise few structural domains, which, in some cases, are not even proper proteins (for example, “small peptides”).

Table 4 shows the frequencies of the four classes in proteins involved in intramolecular DBs. About one-half of them are *all-beta* (50.3% when DBs are identified with the FIND or the GREP methods). These values range from 45.6% to 54.9% (FIND method) and from 45.9% to 54.1% (GREP method) amongst the 14 subsets of the Protein Data Bank (see Table S8 in the Supplementary Materials for further information). The other classes are much less common, being *all-alpha* close to 10%, *alpha-and-beta* close to 15% and *alpha-plus-beta* slightly over 20%.

These values contrast markedly with the distributions observed in Scop, also shown in Table 4: only 17.8% of the folds and 27.2% of the domains are *all-beta* in Scop.

This suggests that intramolecular DBs are considerably more frequent for proteins rich in β-strands and poor in helices, and agrees with the observation reported above that intermolecular DBs often involve β strands.

A similar trend is observed for intermolecular DBs. Table 5 shows the frequency of observations for heteromeric intermolecular DBs—i.e., when the two proteins bridged by a DB have different sequences.

Clearly, the cases where both proteins are in the *all-beta* class prevail: 45.2% and 39.0% when DBs are identified with the FIND and the GREP methods, respectively. These values range from 32.7% to 69.6% (FIND method) and from 29.1% to 60.2% (GREP method) (see Table S9 in the Supplementary Materials for more details).

A second clear signal offered by Table 5 is that intermolecular DBs are mostly made by proteins that belong to the same structural class. Only about 4% of the intermolecular DBs bridge proteins that belong to different structural classes. This is also shown by disaggregated data when the 14 subsets are examined one by one (see Table S9 in the Supplementary Materials for more details).

## 4. Conclusions

The survey of the intermolecular DBs deposited in the Protein Data Bank has demonstrated that intermolecular DBs are infrequent but not very rare.

Some observations can be useful in protein modelling and design. Contrary to intramolecular DBs, intermolecular DBs do not need to be well buried in the protein interior. As a consequence, they are less involved in molecular packing and can assume a conformation closer to the most stable rotamers. This agrees with the fact that, despite them being more accessible to the solvent, they have B-factors similar to the more buried intermolecular DBs [40].

From a folding perspective, intermolecular DBs are problematic—and this is certainly the reason why they are not very frequent—since they can also be formed between proteins that should not be bridged by sulfur–sulfur covalent bonds. However, it is possible to speculate on the existence of a general mechanism to prevent misassembling. Some indications along this way have been observed.

First, heteromeric DBs, which bridge proteins that have different sequences, are more common than homomeric DBs, despite the increased likelihood of incorrect assembly as the number of potential partners increases. Then, the number of intermolecular DBs tends to be slightly larger in heteromeric assemblies, suggesting an evolutionary pressure towards heteromeric DBs. Furthermore, while intramolecular DBs are most observed in hydrolases, heteromeric DBs can be formed in any class of enzymes, suggesting that the general mechanism that prevents misassembling can work on various types of protein and it is not limited to a specific type of proteins.

Periodical updates to the atlas of intermolecular DBs present in this manuscript will be necessary as new information becomes available. This might bring new discoveries, especially by examining structures determined at very high resolution, not only with X-ray crystallography but also with other experimental techniques, such as, for example, cryo-EM or electron diffraction.

## Figures and Tables

**Table 1:** Percentages of observations of the numbers of intermolecular DBs (NSS) between two identical or different protein chains (DBs are detected by the FIND or the GREP method).

	FIND		GREP	
NSS	Identical	Different	Identical	Different
1	83.9 (1.3)	77.3 (1.6)	86.3 (0.8)	76.5 (1.6)
2	15.6 (0.9)	22.3 (1.7)	13.1 (0.7)	23.0 (1.7)
3	0.5 (0.4)	0.5 (0.2)	0.6 (0.3)	0.5 (0.2)

**Table 2:** Percentages of secondary structures (ss) of the cysteines that form intra- or intermolecular disulfide bonds. Secondary structures were assigned with DSSP or Stride. Disulfide bonds were identified with the FIND or the GREP methods. Only data with percentages of observations larger than 5% (in any combination DSSP/Stride or FIND/GREP) are shown. Estimated standard errors are in parentheses.

ss	DSSP				Stride			
	FIND		GREP		FIND		GREP	
	Intra	Inter	Intra	Inter	Intra	Inter	Intra	Inter
**HH**	4.5 (0.2)	9.8 (0.7)	4.6 (0.2)	11.3 (0.6)	6.1 (0.2)	15.1 (1.2)	7.3 (0.7)	11.2 (2.3)
**HE**	5.2 (0.2)	0.2 (0.1)	5.2 (0.2)	0.2 (0.1)	5.3 (0.3)	0.2 (0.1)	7.3 (0.5)	1.6 (1.1)
**EE**	22.7 (0.3)	15.8 (0.7)	22.5 (0.2)	15.2 (0.8)	23.0 (0.4)	14.1 (1.2)	18.2 (0.7)	7.8 (1.2)
**CE**	0.0 (0.0)	0.0 (0.0)	0.0 (0.0)	0.0 (0.0)	6.2 (0.2)	1.2 (0.2)	5.5 (0.3)	1.1 (0.3)
**TH**	1.9 (0.1)	2.5 (0.4)	1.9 (0.1)	2.5 (0.4)	4.0 (0.1)	0.8 (0.2)	5.1 (0.3)	0.9 (0.4)
**TE**	2.0 (0.1)	0.2 (0.1)	1.9 (0.0)	0.2 (0.1)	5.8 (0.2)	0.6 (0.2)	5.1 (0.2)	0.1 (0.1)
**TT**	0.5 (0.0)	2.1 (0.3)	0.5 (0.0)	2.2 (0.3)	6.3 (0.1)	5.0 (0.6)	7.0 (0.3)	5.8 (1.0)

The secondary structure symbols used in DSSP and Stride are H (alpha-helix), I (PI-helix), G (3–10-helix), E (extended conformation), B or b (isolated bridge), C (coil), S (bent), and T (turn).

**Table 3:** Average percentages (estimated standard deviations in parentheses) of various types of proteins (non-enzyme and the seven types of enzymes) that contain both intra- and intermolecular disulfide bonds (Both), only intramolecular disulfide bonds (Intra-only) or only intermolecular disulfide bonds (Inter-only). Disulfide bonds are identified with the FIND or with the GREP method.

Protein	FIND			GREP		
	Both	Intra-Only	Inter-Only	Both	Intra-Only	Inter-Only
Non-enzyme	57.3 (1.0)	47.6 (0.3)	54.2 (2.0)	57.0 (1.0)	47.5 (0.3)	50.9 (1.7)
Oxidoreductases	1.9 (0.2)	6.3 (0.2)	9.8 (0.8)	2.1 (0.2)	7.0 (0.2)	13.5 (1.0)
Transferases	0.8 (0.2)	3.5 (0.2)	14.8 (0.7)	1.5 (0.3)	3.9 (0.2)	13.7 (0.7)
Hydrolases	39.2 (1.0)	41.1 (0.5)	13.5 (1.4)	38.1 (1.0)	39.7 (0.5)	12.4 (1.3)
Lyases	0.7 (0.2)	0.9 (0.1)	3.2 (0.5)	1.2 (0.2)	1.0 (0.1)	3.6 (0.7)
Isomerases	0.0 (0.0)	0.3 (0.0)	2.5 (0.6)	0.0 (0.0)	0.4 (0.0)	2.4 (0.4)
Ligases	0.1 (0.1)	0.3 (0.0)	2.1 (0.6)	0.2 (0.1)	0.4 (0.1)	3.5 (0.6)
Translocases	0.0 (0.0)	0.0 (0.0)	0.0 (0.0)	0.0 (0.0)	0.0 (0.0)	0.0 (0.0)

**Table 4:** Percentages of Scop classes (***a***, ***b***, ***c*** and ***d***) in proteins containing intramolecular DBs identified with the FIND and GREP methods (estimated standard deviations in parentheses). For comparison, the composition of Scop is given (% of folds and % of domains).

Class	FIND	GREP	% of Folds	% of Domains
***a***	11.1 (2.3)	11.1 (2.5)	28.6	15.8
***b***	50.3 (3.7)	50.3 (3.4)	17.8	27.2
***c***	16.0 (2.3)	16.2 (2.3)	14.6	29.9
***d***	22.6 (3.5)	22.4 (3.3)	39.1	27.1

**Table 5:** Percentages of proteins belonging to Scop classes ***a***–***d*** connected to proteins belonging to Scop classes ***a***–***d*** by an intermolecular disulfide bond, identified with the FIND and GREP methods (estimated standard deviations in parentheses). Only heteromeric couples of proteins are considered.

FIND				
Class	*a*	*b*	*c*	*d*
***a***	13.1 (4.7)	0.0 (0.0)	0.4 (0.7)	0.1 (0.4)
***b***	0.0 (0.0)	45.2 (3.9)	0.1 (0.3)	1.4 (1.3)
***c***	0.4 (0.7)	0.1 (0.3)	16.6 (2.6)	0.2 (0.8)
***d***	0.1 (0.4)	1.4 (1.3)	0.2 (0.8)	20.7 (3.0)
**GREP**				
**Class**	***a***	***b***	***c***	***d***
***a***	12.3 (4.1)	0.1 (0.2)	0.3 (0.6)	0.2 (0.4)
***b***	0.1 (0.2)	39.0 (3.1)	0.1 (0.4)	1.3 (1.2)
***c***	0.3 (0.6)	0.1 (0.4)	18.7 (2.4)	0.2 (0.6)
***d***	0.2 (0.4)	1.3 (1.2)	0.2 (0.6)	25.8 (3.9)

## Data Availability

Data are available on request.

## Supplementary Materials

The following supporting information can be downloaded at: https://www.mdpi.com/article/10.3390/life12070986/s1.
