# Supplementary material for: Survey of the Intermolecular Disulfide Bonds Observed in Protein Crystal Structures Deposited in the Protein Data Bank
Source: Life (Basel). 2022 Jun 30;12(7):986. doi: 10.3390/life12070986 (PMC9323673; doi:10.3390/life12070986)
Supplement: Supplementary file 1 [file life-12-00986-s001.zip › life-1787448-supplementary.pdf]

Survey of the intermolecular disulfide bonds observed in protein crystal structures deposited in the Protein Data Bank

Oliviero Carugo

Department of Chemistry, University of Pavia, Italy

Department of Structural and Computational Biology, University of Vienna, Austria (0000-0002-2924-9016)

Correspondence to:

Oliviero Carugo

Department of Chemistry

University of Pavia

viale Taramelli 12

I-27100 Pavia

Italy

oliviero.carugo@univie.ac.at

Comparison between disulfide bonds found from the atomic coordinates and from the SSBOND lines of the PDB files.

The presence of disulfide bonds was detected with two strategies, *FIND* and *GREP*.

On the one hand (*FIND* method), disulfide bonds are detected from the atomic coordinates, under the condition that the S-S covalent bond length is in the 1.9–2.1 Å range (in the case of conformational disorder, only the first conformation is considered).

On the other hand (*GREP* method), the disulfide bonds listed in the "SSBOND" lines of the PDB-formatted files are considered.

Minor differences between the results provided by the two methods are observed in 5.1% of the PDB files (from 4.8% to 5.6% amongst the 14 sets of PDB structures).

Here, a couple of examples of these discrepancies are given.

In 1fgb (cholera toxin B subunit pentamer at 2.40 Å resolution; reference A), there are five disulfide bonds, one in each protomer, between cysteines 9 and 86 (*GREP* method). However, one of them is

not detected from the analysis of the atomic coordinates since it is too long (2.14 Å; chain E), outside of the 1.9–2.1 Å range (*FIND* method).

In 1zr0 (bovine trypsin complexed by tissue factor pathway inhibitor 2 at 1.80 Å resolution; reference B), there are 17 disulfide bonds, six in chain A and in chain C (trypsin), and three in chain C and in chain D (inhibitor) (*GREP* method). However, several of them are longer than 2.1 Å—one is even 2.80 Å long—and are thus outside of the 1.9–2.1 Å range (*FIND* method).

The number of disulfide bonds found with the *FIND* methods is never larger than the number of disulfide bonds found with the *GREP* method.

A.
JRNL AUTH R.G.ZHANG,M.L.WESTBROOK,E.M.WESTBROOK,D.L.SCOTT,
JRNL AUTH 2 Z.OTWINOWSKI,P.R.MAULIK,R.A.REED,G.G.SHIPLEY
JRNL TITL THE 2.4 A CRYSTAL STRUCTURE OF CHOLERA TOXIN B SUBUNIT
JRNL TITL 2 PENTAMER: CHOLERAGENOID.
JRNL REF J.MOL.BIOL. V. 251 550 1995
B.
JRNL AUTH A.E.SCHMIDT,H.S.CHAND,D.CASCIO,W.KISIEL,S.P.BAJAJ
JRNL TITL CRYSTAL STRUCTURE OF KUNITZ DOMAIN 1 (KD1) OF
JRNL TITL 2 TISSUE FACTOR PATHWAY INHIBITOR-2 IN COMPLEX WITH
JRNL TITL 3 TRYPSIN. IMPLICATIONS FOR KD1 SPECIFICITY OF
JRNL TITL 4 INHIBITION
JRNL REF J.BIOL.CHEM. V. 280 27832 2005

Table S1. Number of intramolecular (intra) and intermolecular (inter) disulfide bonds found by the FIND and the GREP methods in the 14 subsets of the Protein Data Bank. The percentages are computed as 100* inter/(intra+inter).

	FIND			GREP		
Set	intra	inter	%	intra	inter	%
1	6656	283	4.08	7328	351	4.57
2	6660	307	4.41	7330	352	4.58
3	7006	306	4.18	7736	363	4.48
4	6844	409	5.64	7617	451	5.59
5	6749	335	4.73	7455	380	4.85
6	6846	260	3.66	7541	324	4.12
7	6851	341	4.74	7599	392	4.91
8	7016	323	4.40	7688	376	4.66
9	6779	320	4.51	7467	375	4.78
10	6332	290	4.38	6988	348	4.74
11	6658	289	4.16	7219	334	4.42
12	6564	315	4.58	7161	366	4.86
13	6670	359	5.11	7353	424	5.45
14	6641	323	4.64	7294	379	4.94

Table S2. Percentages of homomeric and heteromeric intermolecular disulfide bonds found by the FIND and GREP methods in the 14 subsets of the Protein Data Bank. A homomeric disulfide bond connects two proteins that have the same amino acidic sequence; a heteromeric disulfide bond connects two proteins that have different amino acidic sequences.

	FIND		GREP	
Set	homomeric	heteromeric	homomeric	heteromeric
1	32.5	67.5	41.1	58.9
2	27.5	72.5	34.5	65.5
3	35.5	64.5	40.5	59.5
4	34.0	66.0	38.2	61.8
5	28.3	71.7	34.3	65.7
6	30.7	69.3	39.5	60.5
7	40.8	59.2	44.4	55.6
8	33.3	66.7	39.3	60.7
9	25.9	74.1	31.6	68.4
10	32.5	67.5	39.4	60.6
11	34.3	65.7	41.7	58.3
12	34.3	65.7	40.4	59.6
13	23.8	76.2	32.6	67.4
14	33.9	66.1	43.8	56.2

Table S3. Percentages of observations of the numbers of intermolecular DBs (NSS) between two identical or different protein chains (DBs are detected by the FIND or the GREP method) in the 14 subsets of the Protein Data Bank.

Set 1				
	FIND		GREP	
NSS	identical	different	identical	different
1	68.9	84.4	78.0	82.2
2	25.7	15.0	18.6	17.2
3	5.4	0.6	3.4	0.6
Set 2				
	FIND		GREP	
NSS	identical	different	identical	different
1	82.6	75.8	85.0	75.3
2	17.4	24.2	15.0	24.7
3	0.0	0.0	0.0	0.0
Set 3				
	FIND		GREP	
NSS	identical	different	identical	different
1	82.6	83.8	82.9	81.2
2	17.4	14.4	17.1	17.1
3	0.0	1.8	0.0	1.7
Set 4				
	FIND		GREP	
NSS	identical	different	identical	different
1	88.3	67.4	89.2	68.0
2	10.8	32.6	10.1	32.0
3	0.9	0.0	0.7	0.0
Set 5				
	FIND		GREP	
NSS	identical	different	identical	different
1	85.5	72.6	86.8	72.4
2	14.5	26.9	13.2	27.1
3	0.0	0.5	0.0	0.5
Set 6				
	FIND		GREP	
NSS	identical	different	identical	different
1	87.1	85.4	87.6	86.1
2	12.9	14.6	12.4	13.9
3	0.0	0.0	0.0	0.0
Set 7				
	FIND		GREP	
NSS	identical	different	identical	different
1	89.0	77.2	88.4	76.1
2	11.0	22.8	11.6	23.9
3	0.0	0.0	0.0	0.0
Set 8				
	FIND		GREP	
NSS	identical	different	identical	different
1	86.5	76.4	87.8	75.8

2	13.5	23.0	12.2	23.7
3	0.0	0.6	0.0	0.5
Set 9				
	FIND		GREP	
NSS	identical	different	identical	different
1	85.9	83.7	86.1	83.1
2	14.1	14.8	12.9	15.5
3	0.0	1.5	1.0	1.4
Set 10				
	FIND		GREP	
NSS	identical	different	identical	different
1	85.9	77.8	86.7	75.9
2	14.1	20.4	12.4	22.4
3	0.0	1.8	0.9	1.7
Set 11				
	FIND		GREP	
NSS	identical	different	identical	different
1	86.7	78.6	89.8	78.2
2	13.3	20.1	9.3	20.6
3	0.0	1.3	0.8	1.2
Set 12				
	FIND		GREP	
NSS	identical	different	identical	different
1	81.3	81.0	84.9	81.2
2	18.7	19.0	15.1	18.8
3	0.0	0.0	0.0	0.0
Set 13				
	FIND		GREP	
NSS	identical	different	identical	different
1	82.1	70.1	87.2	67.1
2	14.9	29.9	11.0	32.9
3	3.0	0.0	1.8	0.0
Set 14				
	FIND		GREP	
NSS	identical	different	identical	different
1	82.4	67.5	87.2	67.8
2	17.6	31.9	12.8	31.6
3	0.0	0.6	0.0	0.6

Table S4. Percentages of secondary structures (ss) of the cysteines that form intra- or intermolecular DBs in the 14 subsets of the Protein Data Bank. Secondary structure assignments were performed with DSSP or Stride. DBs were identified with the FIND or the GREP methods. Only data with percentages of observations larger than 5% (in any combination DSSP/Stride or FIND/GREP) are shown.

Set 1								
	DSSP				Stride			
	FIND		GREP		FIND		GREP	
ss	Intra	Inter	Intra	Inter	Intra	Inter	Intra	inter
HH	4.2	13.5	5.6	11.7	4.4	15.7	3.1	7.4
HE	6.2	0.0	6.2	0.0	6.2	0.0	11.8	0.0
EE	22.8	16.7	23.2	16.7	22.2	16.0	17.5	3.7
CE	0.0	0.0	7.8	1.4	0.0	0.0	8.1	0.0
TH	1.8	0.0	3.7	1.1	1.9	0.0	4.3	0.0
TE	1.8	0.0	6.0	0.0	1.7	0.0	4.9	0.0
TT	0.3	1.1	5.6	5.0	0.4	2.0	8.8	3.7
Set 2								
	DSSP				Stride			
	FIND		GREP		FIND		GREP	
ss	Intra	Inter	Intra	Inter	Intra	Inter	Intra	inter
HH	5.4	8.1	7.3	14.0	5.3	10.5	7.9	14.4
HE	4.9	0.0	4.9	0.0	5.1	0.0	5.6	0.0
EE	22.9	19.2	23.5	18.2	22.3	18.5	21.2	11.5
CE	0.0	0.0	5.4	0.7	0.0	0.0	5.6	0.8
TH	2.0	3.6	3.9	0.7	1.9	3.7	3.9	1.2
TE	2.2	0.3	6.5	0.3	2.0	0.3	6.0	0.0
TT	0.3	2.0	5.8	4.6	0.3	2.0	6.2	6.6
Set 3								
	DSSP				Stride			
	FIND		GREP		FIND		GREP	
ss	Intra	Inter	Intra	Inter	Intra	Inter	Intra	inter
HH	4.4	9.2	6.2	11.5	4.4	10.2	12.4	2.0
HE	4.7	0.3	4.6	0.0	4.6	0.3	7.2	0.0
EE	23.8	19.3	24.2	18.0	23.3	18.8	13.6	9.8
CE	0.0	0.0	6.6	2.0	0.0	0.0	5.6	2.0
TH	1.7	1.6	3.7	2.3	1.8	2.2	6.0	0.0
TE	2.0	0.3	6.1	0.3	2.0	0.3	2.9	0.0
TT	0.5	1.6	6.2	5.6	0.5	1.4	6.4	5.9
Set 4								
	DSSP				Stride			
	FIND		GREP		FIND		GREP	
ss	Intra	Inter	Intra	Inter	Intra	Inter	Intra	inter
HH	4.9	12.2	6.0	17.3	4.7	12.2	6.9	0.0
HE	5.9	0.0	5.8	0.0	6.0	0.0	7.1	0.0
EE	20.9	15.6	21.6	15.1	20.7	15.9	17.4	16.7
CE	0.0	0.0	6.9	1.2	0.0	0.0	5.5	0.0
TH	1.7	4.4	3.6	1.0	1.6	4.2	6.4	4.2
TE	2.0	1.0	6.1	1.0	2.0	0.9	4.9	0.0
TT	0.3	1.7	6.8	2.9	0.4	1.5	5.9	0.0

Set 5								
	DSSP				Stride			
	FIND		GREP		FIND		GREP	
ss	Intra	Inter	Intra	Inter	Intra	Inter	Intra	inter
HH	3.8	7.2	5.3	15.9	4.0	8.4	5.8	13.2
HE	4.4	0.0	4.3	0.0	4.6	0.0	6.0	0.0
EE	22.4	18.0	22.9	18.0	22.2	17.4	18.4	9.2
CE	0.0	0.0	6.1	0.9	0.0	0.0	5.8	1.3
TH	1.9	5.7	4.2	0.3	1.9	5.3	5.0	1.3
TE	1.8	0.0	5.9	0.9	1.7	0.0	6.6	0.0
TT	0.3	1.5	6.3	4.2	0.3	2.4	7.6	1.3
Set 6								
	DSSP				Stride			
	FIND		GREP		FIND		GREP	
ss	Intra	Inter	Intra	Inter	Intra	Inter	Intra	inter
HH	4.7	6.2	6.2	11.2	4.7	9.9	7.0	15.5
HE	4.5	0.0	4.4	0.0	4.5	0.0	5.9	0.0
EE	22.5	15.8	23.1	15.0	22.5	16.0	19.1	9.0
CE	0.0	0.0	5.8	2.7	0.0	0.0	5.3	3.9
TH	2.0	2.7	3.7	0.0	2.0	2.2	4.1	0.0
TE	2.0	0.0	5.9	1.9	1.9	0.0	5.8	0.0
TT	0.5	2.3	6.1	3.8	0.4	2.2	7.4	3.2
Set 7								
	DSSP				Stride			
	FIND		GREP		FIND		GREP	
ss	Intra	Inter	Intra	Inter	Intra	Inter	Intra	inter
HH	5.1	6.2	6.9	9.7	5.2	7.1	7.7	0.0
HE	5.1	0.0	4.9	0.0	4.9	0.0	7.7	0.0
EE	21.4	12.3	21.9	12.0	21.2	13.5	16.3	4.7
CE	0.0	0.0	5.9	1.2	0.0	0.0	5.3	0.0
TH	2.1	1.8	4.3	1.5	2.1	1.5	6.0	4.7
TE	2.2	0.0	6.1	0.0	2.0	0.0	4.0	0.0
TT	0.5	5.6	6.9	9.7	0.5	5.1	7.1	11.6
Set 8								
	DSSP				Stride			
	FIND		GREP		FIND		GREP	
ss	Intra	Inter	Intra	Inter	Intra	Inter	Intra	inter
HH	4.6	10.2	6.6	13.0	4.6	11.5	11.1	9.8
HE	5.0	1.2	4.9	1.2	5.0	1.1	6.1	9.8
EE	23.3	13.0	23.7	12.7	23.1	13.3	14.4	2.4
CE	0.0	0.0	6.7	0.9	0.0	0.0	5.5	2.4
TH	2.2	1.6	4.2	0.9	2.2	1.9	6.3	0.0
TE	2.0	0.0	6.3	0.9	2.0	0.0	5.3	0.0
TT	0.5	3.1	5.9	6.5	0.5	2.9	5.2	12.2
Set 9								
	DSSP				Stride			
	FIND		GREP		FIND		GREP	
ss	Intra	Inter	Intra	Inter	Intra	Inter	Intra	inter
HH	3.4	9.4	5.3	14.1	3.6	9.9	7.3	0.0
HE	4.9	1.2	4.7	1.2	4.7	1.1	5.3	12.9

EE	23.6	17.5	24.5	17.5	23.4	17.6	18.7	9.7
CE	0.0	0.0	5.2	1.2	0.0	0.0	3.6	3.2
TH	2.3	2.5	4.4	0.6	2.1	2.1	7.7	0.0
TE	1.8	0.3	6.4	0.6	1.8	0.5	4.7	0.0
TT	0.4	1.9	6.4	6.9	0.3	1.9	7.9	9.7
Set 10								
	DSSP				Stride			
	FIND		GREP		FIND		GREP	
ss	Intra	Inter	Intra	Inter	Intra	Inter	Intra	inter
HH	4.3	12.4	6.2	15.5	4.5	14.1	8.4	10.0
HE	4.7	0.0	4.7	0.0	4.7	0.0	8.5	0.0
EE	24.2	15.2	24.7	15.2	23.8	14.4	19.0	10.0
CE	0.0	0.0	6.1	1.7	0.0	0.0	3.0	0.0
TH	2.0	1.4	3.9	1.0	2.0	2.3	4.9	0.0
TE	2.1	0.3	5.1	0.3	1.9	0.3	5.0	0.0
TT	0.7	2.8	6.3	3.8	0.7	2.6	6.2	6.7
Set 11								
	DSSP				Stride			
	FIND		GREP		FIND		GREP	
ss	Intra	Inter	Intra	Inter	Intra	Inter	Intra	inter
HH	3.4	12.1	4.7	15.9	3.7	13.2	3.3	22.6
HE	6.2	0.0	6.0	0.0	5.9	0.0	10.6	0.0
EE	22.8	14.5	23.5	13.8	22.6	15.3	18.2	0.0
CE	0.0	0.0	5.9	0.0	0.0	0.0	6.1	0.0
TH	2.1	1.0	4.3	1.0	2.1	0.9	4.2	0.0
TE	1.9	0.7	5.9	1.0	1.8	0.6	5.0	0.0
TT	0.7	1.7	6.5	6.6	0.7	2.1	9.0	6.5
Set 12								
	DSSP				Stride			
	FIND		GREP		FIND		GREP	
ss	Intra	Inter	Intra	Inter	Intra	Inter	Intra	inter
HH	5.0	11.4	6.6	26.9	5.0	10.9	7.8	14.0
HE	6.0	0.0	7.9	0.0	6.0	0.0	7.0	0.0
EE	21.7	16.8	18.5	3.8	21.4	16.9	18.2	11.8
CE	0.0	0.0	5.8	0.0	0.0	0.0	6.6	0.6
TH	1.8	1.9	4.3	0.0	1.9	1.6	4.0	1.1
TE	2.3	0.0	5.0	0.0	2.2	0.0	6.4	1.1
TT	0.4	1.9	6.8	0.0	0.3	1.6	6.3	6.2
Set 13								
	DSSP				Stride			
	FIND		GREP		FIND		GREP	
ss	Intra	Inter	Intra	Inter	Intra	Inter	Intra	inter
HH	5.5	8.9	6.1	19.7	5.5	10.1	6.5	25.9
HE	5.5	0.0	5.9	0.0	5.7	0.0	7.7	0.0
EE	22.6	12.0	23.6	7.5	22.6	12.0	19.6	3.7
CE	0.0	0.0	5.8	2.1	0.0	0.0	5.3	0.0
TH	1.5	3.6	4.0	0.0	1.5	3.8	4.5	0.0
TE	1.9	0.0	4.8	0.0	2.0	0.0	5.2	0.0
TT	0.7	0.3	6.8	5.0	0.7	0.7	6.7	0.0
Set 14								

	DSSP				Stride			
	FIND		GREP		FIND		GREP	
ss	Intra	Inter	Intra	Inter	Intra	Inter	Intra	inter
HH	4.6	11.8	6.2	11.2	4.8	14.9	6.4	22.4
HE	5.5	0.0	4.4	0.0	5.4	0.0	5.7	0.0
EE	23.1	7.5	23.1	15.0	23.1	7.4	23.3	7.7
CE	0.0	0.0	5.8	2.7	0.0	0.0	6.0	1.7
TH	2.1	3.4	3.7	0.0	2.0	2.9	4.0	0.3
TE	2.1	0.0	5.9	1.9	2.1	0.0	5.2	0.0
TT	0.6	1.6	6.1	3.8	0.6	2.4	6.8	7.3

Table S5. Average values (standard deviations of the mean in parentheses) of the solvent accessible surface area (Å^2) of the cysteines forming intra- or intermolecular disulfide bonds in the 14 subsets of the Protein Data Bank. Disulfide bonds have been identified with the FIND and GRAP methods.

	FIND		GREP	
	intra	inter	intra	inter
Set 1	10.7(0.1)	21.5(0.9)	10.9(0.1)	25.7(1.1)
Set 2	11.2(0.2)	22.7(1.0)	11.3(0.1)	25.2(1.1)
Set 3	11.5(0.2)	25.9(1.2)	11.7(0.1)	28.0(1.1)
Set 4	11.9(0.2)	20.7(0.8)	12.0(0.1)	23.0(0.9)
Set 5	11.7(0.2)	26.8(1.2)	11.7(0.1)	30.1(1.2)
Set 6	11.6(0.1)	28.0(1.5)	11.7(0.1)	30.4(1.3)
Set 7	11.7(0.1)	25.9(1.0)	11.7(0.1)	27.7(1.0)
Set 8	11.4(0.1)	29.8(1.3)	11.4(0.1)	32.4(1.2)
Set 9	11.1(0.1)	25.6(1.2)	11.1(0.1)	29.0(1.2)
Set 10	11.4(0.2)	22.8(1.1)	11.4(0.1)	25.5(1.1)
Set 11	11.5(0.2)	23.7(1.2)	11.5(0.1)	26.0(1.1)
Set 12	11.5(0.2)	23.6(1.1)	11.7(0.1)	26.6(1.1)
Set 13	11.8(0.2)	20.5(0.9)	11.9(0.1)	24.0(0.9)
Set 14	11.9(0.2)	25.2(1.1)	12.0(0.1)	27.6(1.1)

Table S6. Percentages of various types of proteins (non-enzyme and the seven types of enzymes) that contain both intra- and intermolecular disulfide bonds (Both), only intramolecular disulfide bonds (Intra-only) or only intermolecular disulfide bonds (Inter-only), in the 14 subsets of the Protein Data Bank examined in the present manuscript. Disulfide bonds are identified with the FIND or with the GREP method.

Non-enzyme						
Set	FIND			GREP		
	Both	Intra-only	Inter-only	Both	Intra-only	Inter-only
Set 1	53.8	48.2	65.3	54.7	47.8	56.7
Set 2	55.9	48.0	62.2	55.5	47.7	57.9
Set 3	58.7	46.6	38.5	57.2	46.1	35.0
Set 4	61.2	46.3	62.0	60.2	46.5	50.8
Set 5	58.8	46.9	58.7	59.5	47.3	54.5
Set 6	56.1	46.1	53.8	54.5	46.2	52.4
Set 7	56.8	46.6	50.0	56.5	47.2	49.2
Set 8	59.7	47.1	60.8	60.7	47.0	58.7
Set 9	61.7	46.9	55.6	60.9	47.1	50.0
Set 10	61.3	48.6	47.6	60.3	48.4	50.8
Set 11	56.9	47.7	53.6	57.4	47.4	51.4
Set 12	48.1	49.6	53.8	46.7	49.4	52.9
Set 13	58.8	50.3	53.5	59.3	50.7	51.5
Set 14	54.9	47.0	43.5	54.1	46.7	40.8
Oxidoreductases						
Set	FIND			GREP		
	Both	Intra-only	Inter-only	Both	Intra-only	Inter-only
Set 1	1.0	6.5	8.2	1.7	7.1	13.4
Set 2	2.5	7.3	5.4	3.1	7.9	5.3
Set 3	1.7	6.8	11.5	1.4	7.8	13.3
Set 4	0.8	7.2	4.0	0.8	7.9	10.8
Set 5	1.7	6.8	10.9	1.5	7.4	15.2
Set 6	1.9	5.5	12.8	2.5	6.0	15.9
Set 7	2.4	6.4	9.6	2.2	6.6	16.9
Set 8	3.1	5.8	5.9	3.6	6.6	6.7
Set 9	1.6	5.7	11.1	2.0	6.3	14.3
Set 10	1.7	5.9	14.3	2.3	6.3	18.6
Set 11	2.0	5.3	7.1	2.6	6.3	12.5
Set 12	1.9	6.8	13.5	2.5	7.2	17.1
Set 13	0.9	6.2	11.6	0.8	7.0	15.2
Set 14	3.3	5.7	10.9	3.0	7.1	14.1
Transferases						
Set	FIND			GREP		
	Both	Intra-only	Inter-only	Both	Intra-only	Inter-only
Set 1	1.0	2.9	12.2	1.7	3.3	11.9
Set 2	0.0	2.6	16.2	0.0	3.5	15.8
Set 3	0.8	3.5	13.5	1.4	3.8	11.7
Set 4	0.0	3.1	14.0	0.0	3.3	13.8

Set	FIND			GREP		
Set 5	0.0	4.4	15.2	0.8	4.8	12.1
Set 6	0.9	3.4	17.9	2.5	4.1	11.1
Set 7	0.0	3.2	17.3	2.2	3.5	10.8
Set 8	1.6	3.4	15.7	1.4	4.0	18.7
Set 9	3.1	3.3	17.8	4.0	3.6	14.3
Set 10	0.8	4.0	9.5	0.8	4.8	10.2
Set 11	1.0	3.0	14.3	1.7	3.2	15.3
Set 12	0.0	3.9	17.3	1.6	4.7	15.7
Set 13	0.9	4.6	11.6	0.8	4.9	13.6
Set 14	1.6	3.6	15.2	1.5	3.8	16.9
Hydrolases						
Set	FIND			GREP		
	Both	Intra-only	Inter-only	Both	Intra-only	Inter-only
Set 1	44.2	41.0	10.2	41.9	40.2	10.4
Set 2	41.5	39.4	8.1	41.4	38.0	7.0
Set 3	38.0	41.6	25.0	38.4	40.1	23.3
Set 4	37.2	41.8	8.0	38.3	40.4	10.8
Set 5	37.8	40.9	10.9	36.6	39.1	10.6
Set 6	39.3	43.9	10.3	37.2	42.4	9.5
Set 7	40.8	42.0	15.4	38.4	40.7	12.3
Set 8	34.9	42.6	11.8	33.6	41.0	10.7
Set 9	33.6	42.2	11.1	32.5	40.9	17.9
Set 10	36.1	40.1	14.3	35.9	38.7	8.5
Set 11	38.2	42.5	17.9	35.7	41.1	13.9
Set 12	49.1	38.3	9.6	47.5	37.0	7.1
Set 13	38.6	37.2	14.0	37.4	35.3	12.1
Set 14	39.3	41.8	21.7	38.3	40.3	19.7
Lyases						
Set	FIND			GREP		
	Both	Intra-only	Inter-only	Both	Intra-only	Inter-only
Set 1	0.0	0.9	0.0	0.0	0.9	3.0
Set 2	0.0	1.5	5.4	0.0	1.5	8.8
Set 3	0.8	0.5	3.8	0.7	0.7	6.7
Set 4	0.8	1.0	4.0	0.8	1.2	4.6
Set 5	1.7	0.8	2.2	1.5	0.9	3.0
Set 6	0.9	0.6	0.0	1.7	0.8	1.6
Set 7	0.0	1.2	5.8	0.7	1.2	6.2
Set 8	0.8	0.5	2.0	0.7	0.5	1.3
Set 9	0.0	1.1	2.2	0.7	1.1	0.0
Set 10	0.0	0.9	2.4	0.8	0.9	1.7
Set 11	2.0	1.0	1.8	2.6	1.1	1.4
Set 12	0.9	1.1	3.8	1.6	1.2	2.9
Set 13	0.9	1.0	4.7	1.6	1.1	3.0
Set 14	0.8	1.0	6.5	3.0	1.1	5.6
Isomerases						
Set	FIND			GREP		
	Both	Intra-only	Inter-only	Both	Intra-only	Inter-only

Set 1	0.0	0.2	2.0	0.0	0.3	1.5
Set 2	0.0	0.5	2.7	0.0	0.6	1.8
Set 3	0.0	0.5	1.9	0.0	0.5	3.3
Set 4	0.0	0.3	2.0	0.0	0.4	1.5
Set 5	0.0	0.1	0.0	0.0	0.1	1.5
Set 6	0.0	0.2	5.1	0.0	0.4	3.2
Set 7	0.0	0.2	0.0	0.0	0.4	0.0
Set 8	0.0	0.3	2.0	0.0	0.4	1.3
Set 9	0.0	0.4	0.0	0.0	0.4	1.8
Set 10	0.0	0.2	7.1	0.0	0.4	5.1
Set 11	0.0	0.3	5.4	0.0	0.4	4.2
Set 12	0.0	0.3	1.9	0.0	0.3	2.9
Set 13	0.0	0.6	2.3	0.0	0.6	3.0
Set 14	0.0	0.6	2.2	0.0	0.7	2.8

Ligases

Set	FIND			GREP		
	Both	Intra-only	Inter-only	Both	Intra-only	Inter-only
Set 1	0.0	0.2	2.0	0.0	0.3	3.0
Set 2	0.0	0.5	0.0	0.0	0.6	3.5
Set 3	0.0	0.5	5.8	0.7	1.0	6.7
Set 4	0.0	0.2	6.0	0.0	0.4	7.7
Set 5	0.0	0.2	2.2	0.0	0.4	3.0
Set 6	0.9	0.2	0.0	1.7	0.2	6.3
Set 7	0.0	0.4	1.9	0.0	0.6	4.6
Set 8	0.0	0.3	2.0	0.0	0.4	2.7
Set 9	0.0	0.3	2.2	0.0	0.5	1.8
Set 10	0.0	0.3	4.8	0.0	0.5	5.1
Set 11	0.0	0.1	0.0	0.0	0.4	1.4
Set 12	0.0	0.0	0.0	0.0	0.2	1.4
Set 13	0.0	0.2	2.3	0.0	0.4	1.5
Set 14	0.0	0.2	0.0	0.0	0.2	0.0

Translocases

Set	FIND			GREP		
	Both	Intra-only	Inter-only	Both	Intra-only	Inter-only
Set 1	0.0	0.1	0.0	0.0	0.1	0.0
Set 2	0.0	0.2	0.0	0.0	0.1	0.0
Set 3	0.0	0.0	0.0	0.0	0.0	0.0
Set 4	0.0	0.0	0.0	0.0	0.0	0.0
Set 5	0.0	0.0	0.0	0.0	0.0	0.0
Set 6	0.0	0.0	0.0	0.0	0.0	0.0
Set 7	0.0	0.0	0.0	0.0	0.0	0.0
Set 8	0.0	0.0	0.0	0.0	0.0	0.0
Set 9	0.0	0.0	0.0	0.0	0.0	0.0
Set 10	0.0	0.0	0.0	0.0	0.0	0.0
Set 11	0.0	0.1	0.0	0.0	0.1	0.0
Set 12	0.0	0.0	0.0	0.0	0.0	0.0
Set 13	0.0	0.0	0.0	0.0	0.0	0.0
Set 14	0.0	0.0	0.0	0.0	0.0	0.0

Table S7. Frequency of the seven types of enzymes in the Protein Data Bank (data taken on March the 7th, 2022).

Enzyme	*Counts*	*Percentages*
Hydrolases	40,914	37.2%
Transferases	35,252	32.0%
Oxidoreductases	16,895	15.4%
Lyases	8147	7.4%
Isomerases	4053	3.7%
Ligases	3403	3.1%
Translocases	1359	1.2%

Table S8. Percentages of observations, in each subset of the Protein Data Bank, of the a, b, c and d classes of Scop, in proteins with intramolecular disulfide bonds identified with the FIND and the GREP methods.

	FIND				GREP			
	a	b	c	d	a	b	c	d
Set 1	9.1	52.1	13.1	25.6	10.7	51.2	13.5	24.6
Set 2	14.0	51.0	14.3	20.7	13.6	51.2	14.8	20.4
Set 3	11.6	49.4	15.7	23.3	10.9	50.0	16.0	23.1
Set 4	10.4	48.2	16.1	25.4	9.9	49.6	16.3	24.2
Set 5	9.5	50.2	16.2	24.1	9.5	50.5	16.7	23.3
Set 6	11.8	50.3	16.6	21.3	11.4	49.3	17.2	22.1
Set 7	12.3	45.6	19.4	22.7	12.2	45.9	19.4	22.5
Set 8	11.2	49.8	15.1	24.0	11.0	50.1	15.1	23.9
Set 9	8.1	49.3	17.4	25.2	8.4	49.2	17.5	24.9
Set 10	9.5	54.9	14.1	21.6	10.2	54.1	14.4	21.4
Set 11	9.2	52.3	15.7	22.8	9.2	51.9	16.3	22.5
Set 12	13.1	50.5	15.6	20.8	12.8	50.1	16.0	21.2
Set 13	14.4	50.6	16.3	18.7	14.4	51.0	16.2	18.4
Set 14	11.5	49.6	17.8	21.1	11.3	49.8	18.2	20.8

Table S9. Percentages, for each subset of the Protein Data Bank, with which a fold of class a–d is connected to a fold of class a–d by an intermolecular disulfide bond, identified with the FIND and GREP methods. Classes are taken from the Scop database.

Set 1									
class	FIND				class	GREP			
	a	b	c	d		a	b	c	d
a	22.8	0.0	0.0	0.0	a	19.8	0.0	0.0	0.0
b	0.0	44.3	0.0	0.0	b	0.0	35.1	0.9	0.0
c	0.0	0.0	12.7	0.0	c	0.0	0.9	13.5	0.0
d	0.0	0.0	0.0	20.3	d	0.0	0.0	0.0	29.7
Set 2									
class	FIND				class	GREP			
	a	b	c	d		a	b	c	d
a	20.3	0.0	0.0	0.0	a	16.9	0.0	0.0	1.2
b	0.0	40.6	0.0	1.6	b	0.0	33.7	0.0	1.2
c	0.0	0.0	14.1	0.0	c	0.0	0.0	15.7	0.0
d	0.0	1.6	0.0	21.9	d	1.2	1.2	0.0	28.9
Set 3									
class	FIND				class	GREP			
	a	b	c	d		a	b	c	d
a	16.8	0.0	0.0	0.0	a	15.7	0.0	0.0	0.0
b	0.0	32.7	0.0	2.0	b	0.0	29.1	0.0	1.6
c	0.0	0.0	16.8	0.0	c	0.0	0.0	15.7	0.0
d	0.0	2.0	0.0	29.7	d	0.0	1.6	0.0	36.2
Set 4									
class	FIND				class	GREP			
	a	b	c	d		a	b	c	d
a	7.4	0.0	0.0	0.0	a	7.2	0.0	0.0	0.0
b	0.0	44.1	0.0	1.5	b	0.0	38.6	0.0	1.2
c	0.0	0.0	20.6	2.9	c	0.0	0.0	20.5	2.4
d	0.0	1.5	2.9	19.1	d	0.0	1.2	2.4	26.5
Set 5									
class	FIND				class	GREP			
	a	b	c	d		a	b	c	d
a	13.6	0.0	0.0	0.0	a	10.5	0.0	0.0	0.0
b	0.0	47.5	0.0	1.7	b	0.0	38.2	0.0	1.3
c	0.0	0.0	22.0	0.0	c	0.0	0.0	25.0	0.0
d	0.0	1.7	0.0	13.6	d	0.0	1.3	0.0	23.7
Set 6									
class	FIND				class	GREP			
	a	b	c	d		a	b	c	d
a	5.7	0.0	0.0	0.0	a	6.7	0.0	0.0	0.0
b	0.0	41.4	0.0	4.3	b	0.0	36.5	0.0	3.8
c	0.0	0.0	18.6	0.0	c	0.0	0.0	20.2	0.0
d	0.0	4.3	0.0	25.7	d	0.0	3.8	0.0	28.8
Set 7									
class	FIND				class	GREP			
	a	b	c	d		a	b	c	d
a	14.1	0.0	0.0	0.0	a	12.1	0.0	0.0	0.0

b	0.0	39.4	0.0	4.0	b	0.0	37.1	0.0	3.4
c	0.0	0.0	12.1	0.0	c	0.0	0.0	16.4	0.0
d	0.0	4.0	0.0	26.3	d	0.0	3.4	0.0	27.6
Set 8									
class	FIND				class	GREP			
	a	b	c	d		a	b	c	d
a	9.6	0.0	0.0	0.0	a	12.4	0.0	0.0	0.0
b	0.0	42.2	1.2	0.0	b	0.0	35.2	1.0	0.0
c	0.0	1.2	20.5	0.0	c	0.0	1.0	22.9	0.0
d	0.0	0.0	0.0	25.3	d	0.0	0.0	0.0	27.6
Set 9									
class	FIND				class	GREP			
	a	b	c	d		a	b	c	d
a	8.7	0.0	0.0	0.0	a	8.6	0.0	0.0	0.0
b	0.0	69.6	0.0	0.0	b	0.0	60.2	0.0	0.0
c	0.0	0.0	7.2	0.0	c	0.0	0.0	9.7	0.0
d	0.0	0.0	0.0	14.5	d	0.0	0.0	0.0	21.5
Set 10									
class	FIND				class	GREP			
	a	b	c	d		a	b	c	d
a	18.9	0.0	0.0	1.4	a	19.1	0.9	0.0	0.9
b	0.0	36.5	0.0	1.4	b	0.9	30.9	0.0	0.9
c	0.0	0.0	24.3	0.0	c	0.0	0.0	26.4	0.0
d	1.4	1.4	0.0	14.9	d	0.9	0.9	0.0	18.2
Set 11									
class	FIND				class	GREP			
	a	b	c	d		a	b	c	d
a	4.5	0.0	1.5	0.0	a	7.8	0.0	1.1	0.0
b	0.0	59.1	0.0	0.0	b	0.0	47.8	0.0	0.0
c	1.5	0.0	12.1	0.0	c	1.1	0.0	16.7	0.0
d	0.0	0.0	0.0	21.2	d	0.0	0.0	0.0	25.6
Set 12									
class	FIND				class	GREP			
	a	b	c	d		a	b	c	d
a	6.1	0.0	1.5	0.0	a	4.7	0.0	1.2	0.0
b	0.0	47.0	0.0	1.5	b	0.0	42.4	0.0	3.5
c	1.5	0.0	24.2	0.0	c	1.2	0.0	25.9	0.0
d	0.0	1.5	0.0	16.7	d	0.0	3.5	0.0	17.6
Set 13									
class	FIND				class	GREP			
	a	b	c	d		a	b	c	d
a	16.9	0.0	1.4	0.0	a	15.1	0.0	1.1	0.0
b	0.0	42.3	0.0	0.0	b	0.0	41.9	0.0	0.0
c	1.4	0.0	15.5	0.0	c	1.1	0.0	17.2	0.0
d	0.0	0.0	0.0	22.5	d	0.0	0.0	0.0	23.7
Set 14									
class	FIND				class	GREP			
	a	b	c	d		a	b	c	d
a	18.5	0.0	1.2	0.0	a	15.5	0.0	0.9	0.0
b	0.0	46.9	0.0	1.2	b	0.0	39.1	0.0	0.9

c	1.2	0.0	11.1	0.0	c	0.9	0.0	16.4	0.0
d	0.0	1.2	0.0	18.5	d	0.0	0.9	0.0	25.5

(a)

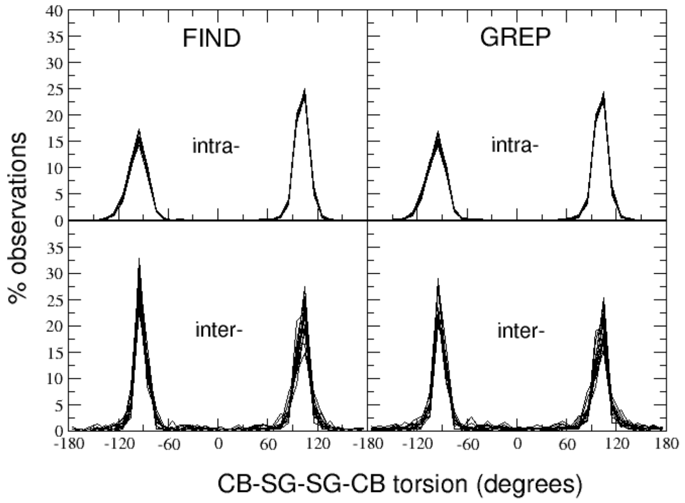

(b)

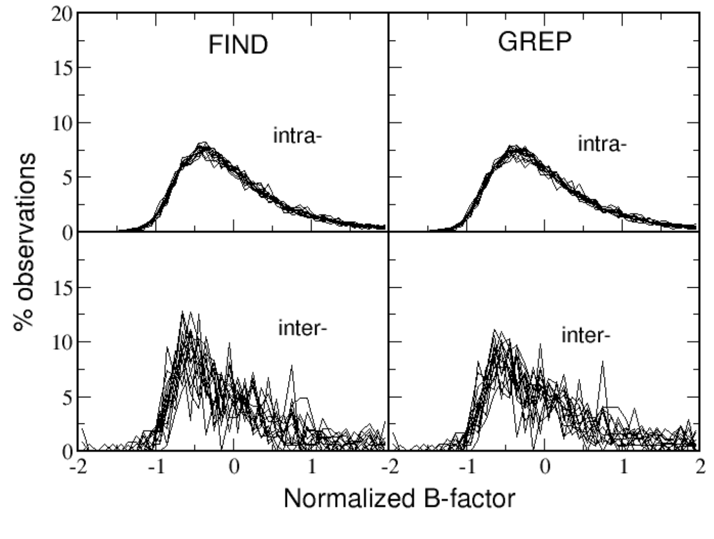

Figure S1. (a) Distribution, for each subset of the Protein Data Bank, of the torsions defined by the CB-SG-SG-CB atoms of the sidechains of the cysteines that form the intra- or intermolecular DBs, identified with the FIND or GREP method; (b) distribution, for each subset of the Protein Data Bank, of the normalized B-factors of the sulfur atoms of the cysteines that form the intra- or intermolecular DBs, identified with the FIND or GREP method.
